# Association between glioma and neurodegenerative diseases risk: a two-sample bi-directional Mendelian randomization analysis

**DOI:** 10.3389/fneur.2024.1413015

**Published:** 2024-07-02

**Authors:** Yang Liu, Youqi Chen, Ming Gao, Jia Luo, Yanan Wang, Yihan Wang, Yu Gao, Laiyu Yang, Jingning Wang, Ningxin Wang

**Affiliations:** ^1^Department of Endocrinology, Affiliated Hospital of Jilin Medical University, Jilin, China; ^2^Bethune First Hospital of Jilin University, Changchun, China; ^3^Bethune Third Hospital of Jilin University, Changchun, China; ^4^Clinical College, The Second Affiliated Hospital of Harbin Medical University, Harbin, China

**Keywords:** Mendelian randomization, neurodegenerative diseases, glioma, genetics, Alzheimer’s disease, multiple sclerosis

## Abstract

**Background:**

Earlier observational studies have demonstrated a correlation between glioma and the risk of neurodegenerative diseases (NDs), but the causality and direction of their associations remain unclear. The objective of this study was to ascertain the causal link between glioma and NDs using Mendelian randomization (MR) methodology.

**Methods:**

Genome-wide association study (GWAS) data were used in a two-sample bi-directional MR analysis. From the largest meta-analysis GWAS, encompassing 18,169 controls and 12,488 cases, summary statistics data on gliomas was extracted. Summarized statistics for NDs, including Alzheimer’s disease (AD), multiple sclerosis (MS), amyotrophic lateral sclerosis (ALS) and Parkinson’s disease (PD) were obtained from the GWAS of European ancestry. Inverse variance weighted (IVW) method was elected as the core MR approach with weighted median (WM) method and MR-Egger method as complementary methods. In addition, sensitivity analyses were performed. A Bonferroni correction was used to correct the results.

**Results:**

Genetically predicted glioma had been related to decreased risk of AD. Specifically, for all glioma (IVW: OR = 0.93, 95% CI = 0.90–0.96, *p* = 4.88 × 10^−6^) and glioblastoma (GBM) (IVW: OR = 0.93, 95% CI = 0.91–0.95, *p* = 5.11 × 10^−9^). We also found that genetically predicted all glioma has a suggestive causative association with MS (IVW: OR = 0.90, 95% CI = 0.81–1.00, *p* = 0.045). There was no evidence of causal association between glioma and ALS or PD. According to the results of reverse MR analysis, no discernible causal connection of NDs was found on glioma. Sensitivity analyses validated the robustness of the above associations.

**Conclusion:**

We report evidence in support of potential causal associations of different glioma subtypes with AD and MS. More studies are required to uncover the underlying mechanisms of these findings.

## Introduction

NDs are major health challenges that have drawn tremendous focus in the previous few years. NDs are an assembly of heterogeneous neurological disorders that involve progressive neuronal damage and death, such as AD, PD, Huntington’s disease, MS and ALS (1). These illnesses significantly diminish patients’ quality of life and frequently result in mortality (2). The etiology of NDs is complex and not well understood. Currently, treatments for NDs have limited effectiveness, with no existing cures available (3). And the prognosis for individuals afflicted with NDs is typically bleak (4). Further research is imperative to enhance the understanding of the underlying causes of NDs and to advance the development of more efficacious treatments.

Gliomas are primary brain tumors that account for approximately 80% of all malignant brain tumors (5). Their 5-year survival rate is less than 20%, indicating a terrible prognosis and imposing a significant health burden on patients (6). Gliomas are divided into different histologic subtypes, the most common and aggressive of which is GBM, which has a 5-year survival rate of only 6.8% (7). The cost of treating individuals with glioma is anticipated to be considerable. Besides, the etiology of glioma is poorly understood.

The relationship between NDs and glioma has been studied to some extent, yet the results remain uncertain. In recent years, increasing prospective studies have supported the correlation between glioma and NDs (8). For instance, several clinical findings imply that glioma growth and progression may be initiated by pre-existing MS or may be facilitated by it (9). Besides, there are more similarities between NDs and brain cancer than previously thought. In addition to having comparable epidemiological and molecular characteristics, these illnesses are associated with some risk factors, such as aging and inflammation (10). Notably, many observational studies may have limitations due to sample numbers and potential confounding variables (11). Therefore, it’s uncertain how NDs and the risk of glioma causally relate.

Utilizing genetic variants as instrumental variables (IVs) to infer the causal relationships between exposures and diseases, MR is a potent approach (12). By exploiting the random assignment of genes during meiosis, MR can circumvent some of the biases inherent in observational studies, such as confusion and inverse causality (13).

In the current research, we implemented a two-sample MR framework to explore the potential role of different glioma subtypes (all glioma, GBM, non-GBM) in the development of four NDs, including AD, MS, ALS, and PD. In addition, we conducted bidirectional MR using gliomas as outcomes to test the direction of association.

## Methods

### Fundamental MR principles

**Figure 1:**
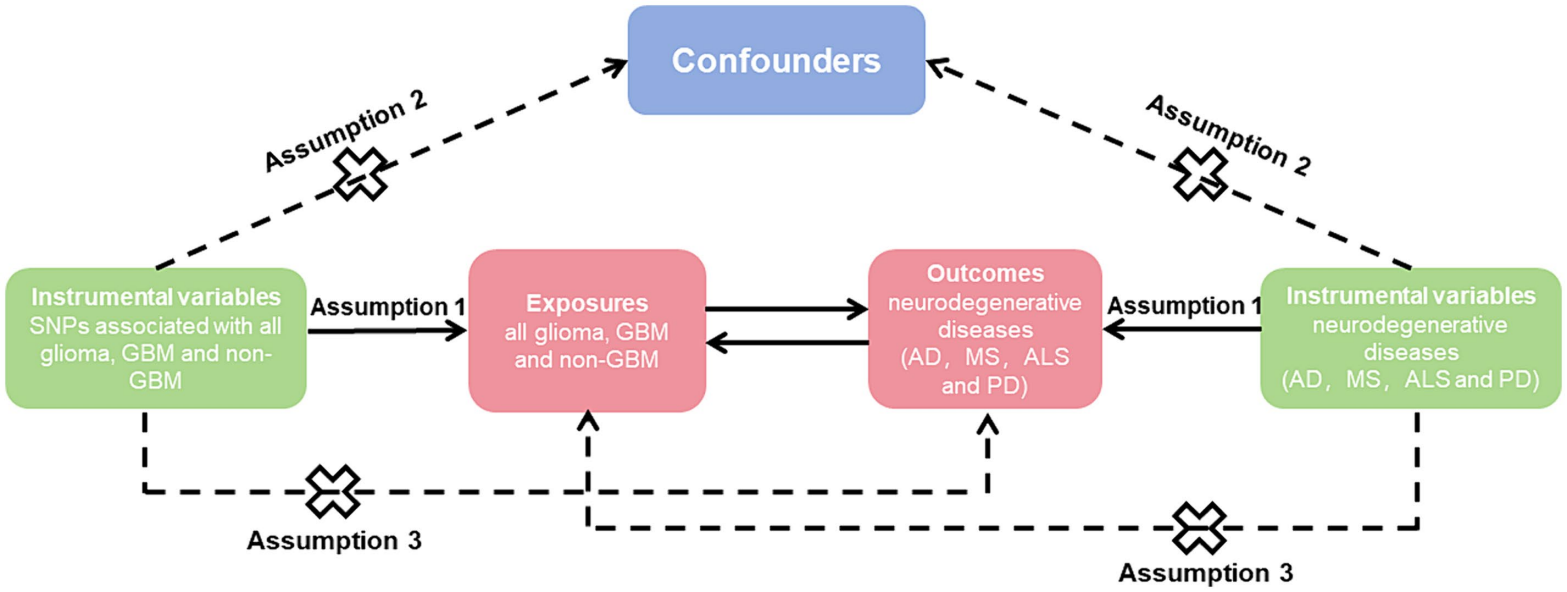
Mendelian randomization causal diagram with three assumptions. (1) Instrumental variables (IVs) must be associated with exposure (*p* < 5 × 10^−8^); (2) IVs are not associated with confounders of the association between exposure and outcome; (3) IVs have no direct effect on the outcome, except through exposure. SNP, single nucleotide polymorphisms; GBM, glioblastoma; AD, Alzheimer’s disease; MS, multiple sclerosis; ALS, amyotrophic lateral sclerosis; PD, Parkinson’s disease.

To explore the causal impact of the risk factor with a desired result, we employed genetic variants as IVs (Figure 1). The study followed three fundamental principles, including: (1) associated with exposure, (2) not associated with confounders of the association between exposure and outcome, and (3) only associated with outcome via their association with exposure (14).

### NDs and glioma GWAS dataset

The two-sample MR studies assumed that the separate samples were used for the exposure and outcome. Therefore, the GWAS summarized data of NDs that had a significant overlap with glioma phenotypes were eliminated. The GWAS summarized data for glioma were obtained from the strongest meta-analysis GWAS, which included 6,183 individuals with glioblastoma multiforme (GBM), 5,820 individuals with non-GBM, and 18,169 European ancestry controls from eight unique GWAS databases (15). The International Multiple Sclerosis Genetics Consortium (IMSGC) provided general information for MS which involves 9,772 cases and 17,376 controls of European descent (16). Summary statistics of PD, AD and ALS were derived from different GWAS. Summary statistics of PD contains 294 cases and 456,054 controls of European ancestry (17). Summary statistics of AD comprise 21,982 late-onset AD individuals with European ancestry, 53,042 European ancestry individuals with a family history of AD, and 397,844 controls with European ancestry (18). Summary statistics of ALS covers 12,577 cases and 23,475 controls (19).

### MR analysis

**Figure 2:**
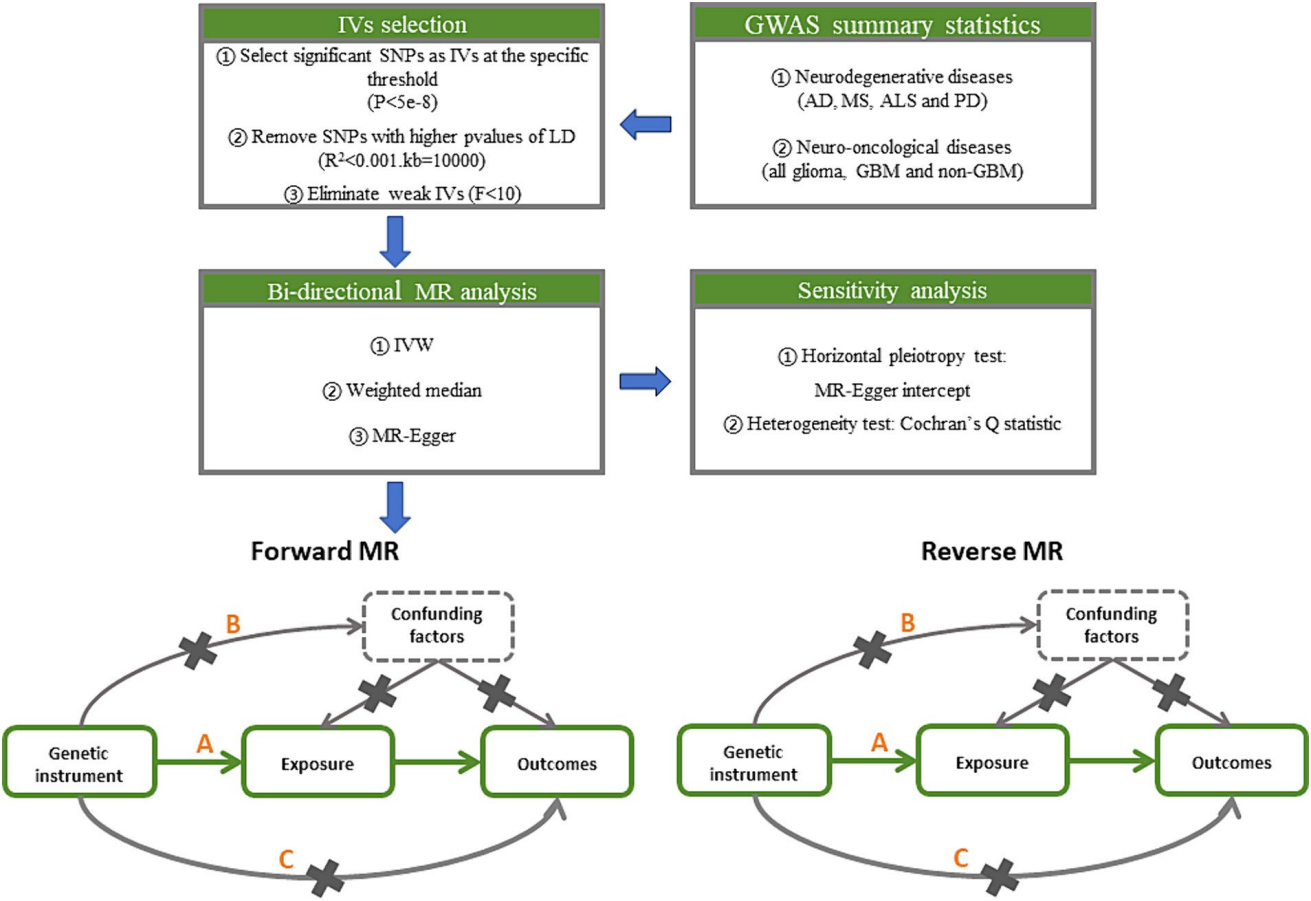
Study design pipeline of the two-sample bi-directional Mendelian randomization analysis. GWAS, Genome-wide association study; AD, Alzheimer’s disease; MS, multiple sclerosis; ALS, amyotrophic lateral sclerosis; PD, Parkinson’s disease; GBM, glioblastoma; IV, instrumental variables; SNP, single nucleotide polymorphisms; LD, linkage disequilibrium; IVW, inverse variance weighted.

To generate IVs, statistically significant threshold [*p* < 5 × 10^−8^; linkage disequilibrium (LD) *r*^2^ < 0.001, LD distance > 10,000 kb] was set (20). For PD, there was no IV’s *p*-value less than 5 × 10^−8^, so we relaxed it to 5 × 10^−6^ (21). We used the IVW approach as the principal analytical approach. The IVW method is a statistical technique used in meta-analysis to combine the results of multiple studies (22). It is based on the principle that studies with larger sample sizes and lower variance should be given more weight in the analysis. We also made reference to the outcomes of other types of models, such as MR-Egger (23) and weighted median (24). A minimum of 50% of the single nucleotide polymorphisms (SNPs) are assumed to be legitimate using the weighted median technique, which provides consistent causal estimates under this assumption. A random effects model may be applied because of the significant heterogeneity among the analyses (Figure 2) (25). Bonferroni correction method was used to adjust the significance level of hypothesis testing (26). If the IVW and weighted median approaches yield consistent results for the direction as well as the magnitude of the causal effects, the presence of causality is indicated (27) and the *p* values of Bonferroni correcting are less than 2.08E^−3^ (0.05/24). *p* values less than 0.05 but greater than 2.08E^−3^ are interpreted as suggestive of a causal relationship.

### Sensitivity analyses

Additionally, in MR studies, horizontal pleiotropy and outlier SNPs can be detected using MR-Egger intercept (28). There are no horizontal pleiotropy effects present if the *p* value of the MR-Egger intercept is higher than 0.05. The heterogeneity between SNPs can be determined using Cochran *Q* statistics, which will guarantee the validity of the MR analysis (29). The strength of genetic instruments can be measured using *F*-statistics, which is calculated using the sample size, number of SNPs, and *R*-squared value (30). A low *F*-statistics value (less than 10) may indicate weak instrument bias (31). The analysis can be performed using the “Two Sample MR” package in R software (32).

## Results

Table 1 displays the enrolled GWAS studies’ summary data. To sum up, 5 GWAS studies (4 GWAS of neurodegenerative diseases and 1 GWAS of glioma) were enrolled in this MR study.

**Table 1:** A brief description of each GWAS summary statistics.

Exposure/outcome	Ancestry	Sample size	Controls	Cases	Year	PubMed ID
All glioma	European	30,657	18,169	12,488	2017	28346443
Glioblastoma (GBM)	European	24,352	18,169	6,183	2017	28346443
Non-GBM	European	23,989	18,169	5,820	2017	28346443
Alzheimer’s disease (AD)	European	472,868	397,844	75,024	2021	33589840
Multiple sclerosis (MS)	European	27,098	17,376	9,722	2011	21833088
Amyotrophic lateral sclerosis (ALS)	European	36,052	23,475	12,577	2016	27455348
Parkinson’s disease (PD)	European	456,348	456,054	294	2021	34737426

### Causal association of glioma on NDs

There were 12–18 SNPs used for MR assessments in the initial analyzing. All SNPs were considered robust since their *F* statistics were all greater than the threshold of 10 (Supplementary Table 1). The results of the MR evaluation and the sensitivity analysis of causality of glioma on NDs are displayed in Tables 2A, 2B, 2C and Supplementary Tables 2, 3.

**Table 2A:** Mendelian randomization estimates, heterogeneity test and pleiotropy test of all glioma on neurodegenerative diseases.

Exposure	Outcome	Method	nSNPs	Beta	OR (95% CI)	*p*	*p* (heterogeneity)	*p* (pleiotropy)
All glioma	AD	IVW	12	−0.07	0.93 (0.90–0.96)	4.88E-06	0.21	0.34
		WM	12	−0.07	0.93 (0.90–0.97)	3.30E-04	0.20	
		MR Egger	12	−0.05	0.95 (0.90–1.01)	0.17		
All glioma	ALS	IVW	12	−0.02	0.99 (0.94–1.04)	0.56	0.69	0.19
		WM	12	−0.01	0.99 (0.93–1.06)	0.80	0.59	
		MR Egger	12	0.05	1.05 (0.95–1.16)	0.38		
All glioma	MS	IVW	4	−0.11	0.90 (0.81–1.00)	0.045	0.40	0.26
		WM	4	−0.35	0.71 (0.51–0.97)	0.17	0.24	
		MR Egger	4	−0.14	0.87 (0.78–0.96)	0.01		
All glioma	PD	IVW	12	0.11	1.12 (0.89–1.41)	0.33	0.53	0.55
		WM	12	0.08	1.08 (0.79–1.48)	0.62	0.58	
		MR Egger	12	−0.01	0.99 (0.64–1.55)	0.98		

nSNPs, number of single nucleotide polymorphisms; *p* (heterogeneity), *p* value of Cochrane’s *Q* value in heterogeneity test; *p* (pleiotropy), *p* value of MR–Egger intercept; AD, Alzheimer’s disease; IVW, inverse variance weighted; WM, weighted median; ALS, amyotrophic lateral sclerosis; MS, multiple sclerosis; PD, Parkinson’s disease.

**Table 2B:** Mendelian randomization estimates, heterogeneity test and pleiotropy test of GBM on neurodegenerative diseases.

Exposure	Outcome	Method	nSNPs	Beta	OR (95% CI)	*p*	*p* (heterogeneity)	*p* (pleiotropy)
GBM	AD	IVW	10	−0.07	0.93 (0.91–0.95)	5.11E-09	0.40	0.79
		WM	10	−0.06	0.94 (0.91–0.97)	9.32E-05	0.50	
		MR Egger	10	−0.08	0.92 (0.86–0.98)	0.04		
GBM	ALS	IVW	10	4.50E-03	1.00 (0.96–1.05)	0.85	0.88	0.91
		WM	10	−3.30E-03	1.00 (0.94–1.05)	0.91	0.92	
		MR Egger	10	−2.02E-03	1.00 (0.89–1.12)	0.97		
GBM	MS	IVW	3	−0.07	0.93 (0.83–1.04)	0.22	0.15	0.56
		WM	3	−0.20	0.82 (0.60–1.13)	0.44	0.17	
		MR Egger	3	−0.09	0.91 (0.83–1.01)	0.07		
GBM	PD	IVW	10	0.07	1.07 (0.86–1.34)	0.54	0.28	0.62
		WM	10	0.08	1.08 (0.83–1.43)	0.56	0.34	
		MR Egger	10	0.21	1.24 (0.68–2.24)	0.50		

nSNPs, number of single nucleotide polymorphisms; *p* (heterogeneity), *p* value of Cochrane’s *Q* value in heterogeneity test; *p* (pleiotropy), *p* value of MR–Egger intercept; GBM, glioblastoma; AD, Alzheimer’s disease; IVW, inverse variance weighted; WM, weighted median; ALS, amyotrophic lateral sclerosis; MS, multiple sclerosis; PD, Parkinson’s disease.

**Table 2C:** Mendelian randomization estimates, heterogeneity test and pleiotropy test of non-GBM on neurodegenerative diseases.

Exposure	Outcome	Method	nSNPs	Beta	OR (95% CI)	*p*	*p* (heterogeneity)	*p* (pleiotropy)
Non-GBM	AD	IVW	15	−0.04	0.96 (0.93–1.00)	0.03	0.01	0.33
		WM	15	−0.01	0.99 (0.96–1.03)	0.65	0.01	
		MR Egger	15	−0.02	0.98 (0.93–1.04)	0.61		
Non-GBM	ALS	IVW	15	−0.03	0.97 (0.93–1.02)	0.23	0.95	0.22
		WM	15	−0.01	0.99 (0.94–1.05)	0.76	0.90	
		MR Egger	15	0.01	1.01 (0.94–1.09)	0.77		
Non-GBM	MS	IVW	6	−0.10	0.90 (0.81–1.00)	0.05	0.28	0.56
		WM	6	0.05	1.06 (0.64–1.75)	0.84	0.35	
		MR Egger	6	−0.09	0.91 (0.80–1.03)	0.14		
Non-GBM	PD	IVW	15	0.04	1.04 (0.86–1.27)	0.67	0.75	0.99
		WM	15	0.04	1.04 (0.81–1.34)	0.74	0.81	
		MR Egger	15	0.04	1.04 (0.76–1.42)	0.80		

nSNPs, number of single nucleotide polymorphisms; *p* (heterogeneity), *p* value of Cochrane’s *Q* value in heterogeneity test; *p* (pleiotropy), *p* value of MR–Egger intercept; GBM, glioblastoma; AD, Alzheimer’s disease; IVW, inverse variance weighted; WM, weighted median; ALS, amyotrophic lateral sclerosis; MS, multiple sclerosis; PD, Parkinson’s disease.

Our research revealed a strong causal link between AD and genetically predicted gliomas. Specifically, for all glioma (IVW: OR = 0.93, 95% CI = 0.90–0.96, *p* = 4.88 × 10^−6^; WM: OR = 0.93, 95% CI = 0.90–0.97, *p* = 3.30 × 10^−4^) and GBM (IVW: OR = 0.93, 95% CI = 0.91–0.95, *p* = 5.11 × 10^−9^; WM: OR = 0.94, 95% CI = 0.91–0.97, *p* = 9.32 × 10^−5^). It is noteworthy that a suggestive causative association between genetically predicted all glioma and MS was discovered (*p*_adj_ > 0.05 and IVW *p* < 0.05) and genetically predicted non-GBM appeared suggestively to be causally related to AD (*p*_adj_ > 0.05 and IVW *p* < 0.05). Furthermore, a link between glioma and the likelihood of developing ALS and PD was not found (IVW *p* > 0.05) (Tables 2A, 2B, 2C).

### Causal association of NDs on glioma

We designated the SNPs linked to NDs as exposure IVs to evaluate the link of causality between NDs and glioma. Four to forty SNPs were utilized in the MR calculations. All SNPs were robust since their *F* values were much higher than the cutoff of 10 (Supplementary Table 4). A summary is provided in Tables 3A, 3B and 3C and Supplementary Tables 5, 6 of the results obtained from the MR analysis and sensitivity analysis exploring the causative association between NDs and gliomas.

**Table 3A:** Mendelian randomization estimates, heterogeneity test and pleiotropy test of neurodegenerative diseases on all glioma.

Exposure	Outcome	Method	nSNPs	Beta	OR (95% CI)	*p*	*p* (heterogeneity)	*p* (pleiotropy)
AD	All glioma	IVW	29	0.01	1.01 (0.94–1.09)	0.75	0.17	0.38
		WM	29	−0.02	0.98 (0.89–1.08)	0.70	0.17	
		MR Egger	29	−0.03	0.97 (0.86–1.10)	0.64		
ALS	All glioma	IVW	3	0.10	1.10 (0.92–1.32)	0.28	0.77	1.00
		WM	3	0.11	1.11 (0.92–1.35)	0.27	0.96	
		MR Egger	3	0.10	1.11 (0.50–2.46)	0.85		
MS	All glioma	IVW	25	−0.01	0.99 (0.93–1.05)	0.75	0.02	0.97
		WM	25	−0.01	0.99 (0.89–1.10)	0.84	0.02	
		MR Egger	25	−0.02	0.98 (0.92–1.05)	0.63		
PD	All glioma	IVW	5	−0.01	0.99 (0.94–1.04)	0.63	0.80	0.12
		WM	5	−0.01	0.99 (0.94–1.05)	0.77	0.24	
		MR Egger	5	−0.10	0.91 (0.83–0.99)	0.12		

nSNPs, number of single nucleotide polymorphisms; *p* (heterogeneity), *p* value of Cochrane’s *Q* value in heterogeneity test; *p* (pleiotropy), *p* value of MR–Egger intercept; AD, Alzheimer’s disease; IVW, inverse variance weighted; WM, weighted median; ALS, amyotrophic lateral sclerosis; MS, multiple sclerosis; PD, Parkinson’s disease.

**Table 3B:** Mendelian randomization estimates, heterogeneity test and pleiotropy test of neurodegenerative diseases on GBM.

Exposure	Outcome	Method	nSNPs	Beta	OR (95% CI)	*p*	*p* (heterogeneity)	*p* (pleiotropy)
AD	GBM	IVW	29	0.03	1.03 (0.93–1.13)	0.61	0.08	0.54
		WM	29	0.02	1.02 (0.90–1.15)	0.79	0.09	
		MR Egger	29	−0.01	0.99 (0.85–1.15)	0.89		
ALS	GBM	IVW	3	0.08	1.08 (0.87–1.35)	0.48	0.92	0.79
		WM	3	0.09	1.09 (0.86–1.38)	0.47	0.94	
		MR Egger	3	−0.09	0.91 (0.34–2.43)	0.88		
MS	GBM	IVW	24	−0.01	0.99 (0.92–1.06)	0.79	0.04	0.92
		WM	24	−0.01	0.99 (0.87–1.12)	0.82	0.05	
		MR Egger	24	−0.03	0.97 (0.90–1.05)	0.42		
PD	GBM	IVW	4	0.04	1.04 (0.97–1.10)	0.25	0.18	0.41
		WM	4	0.02	1.02 (0.95–1.10)	0.51	0.17	
		MR Egger	4	−0.07	0.93 (0.64–1.35)	0.74		

nSNPs, number of single nucleotide polymorphisms; *p* (heterogeneity), *p* value of Cochrane’s *Q* value in heterogeneity test; *p* (pleiotropy), *p* value of MR–Egger intercept; AD, Alzheimer’s disease; GBM, glioblastoma; IVW, inverse variance weighted; WM, weighted median; ALS, amyotrophic lateral sclerosis; MS, multiple sclerosis; PD, Parkinson’s disease.

**Table 3C:** Mendelian randomization estimates, heterogeneity test and pleiotropy test of neurodegenerative diseases on non-GBM.

Exposure	Outcome	Method	nSNPs	Beta	OR (95% CI)	*p*	*p* (heterogeneity)	*p* (pleiotropy)
AD	Non-GBM	IVW	29	0.00	1.00 (0.91–1.09)	0.96	0.71	0.27
		WM	29	−0.10	0.91 (0.80–1.04)	0.16	0.69	
		MR Egger	29	−0.06	0.94 (0.81–1.08)	0.38		
ALS	Non-GBM	IVW	3	0.06	1.07 (0.84–1.35)	0.60	0.53	0.70
		WM	3	0.08	1.08 (0.84–1.39)	0.54	0.72	
		MR Egger	3	0.33	1.39 (0.49–3.98)	0.65		
MS	Non-GBM	IVW	25	−0.03	0.97 (0.91–1.04)	0.46	0.13	0.59
		WM	25	−0.06	0.95 (0.83–1.07)	0.40	0.15	
		MR Egger	25	−0.04	0.96 (0.88–1.05)	0.39		
PD	Non-GBM	IVW	5	−0.01	0.99 (0.93–1.05)	0.75	0.60	0.63
		WM	5	−0.03	0.97 (0.91–1.04)	0.36	0.72	
		MR Egger	5	−0.07	0.93 (0.81–1.07)	0.39		

nSNPs, number of single nucleotide polymorphisms; *p* (heterogeneity), *p* value of Cochrane’s *Q* value in heterogeneity test; *p* (pleiotropy), *p* value of MR–Egger intercept; AD, Alzheimer’s disease; GBM, glioblastoma; IVW, inverse variance weighted; WM, weighted median; ALS, amyotrophic lateral sclerosis; MS, multiple sclerosis; PD, Parkinson’s disease.

NDs with the risk of GBM, non-GBM, and all glioma did not show any causal relationships (*p* > 0.05) (Tables 3A, 3B and 3C). Pleiotropy analyses showed that our MR results had no horizontal pleiotropy (Supplementary Table 6).

## Discussion

NDs are a group of neurological disorders that affect the lives of millions of people worldwide (33). Despite extensive research, the etiology of NDs remains unclear (34). There are several observational studies exploring the causal relationship between NDs and gliomas (8). Due to small sample sizes and inherent biases, establishing causality is difficult (35). We used MR analysis in the present study to investigate the causality and direction of association between different subtypes of glioma and AD, MS, ALS and PD. In this comprehensive analysis of gliomas with risk of NDs, we observed that genetically predicted all glioma and GBM has significant causality with lower risk of AD. Our results also indicate some evidence in favor of a potentially causative link between all glioma and MS, although the association was not survived correction for multiple testing.

Although some research has indicated that the risk of glioma changes possibly during the AD progress, it is unclear what biological mechanism glioma may use to defend against AD (36). A variety of NDs can impact the central nervous system (CNS). Among NDs, AD is the most prevalent (37). Recognizing a wide range of potential threats to the CNS, microglia, which are the main instinctive immune cells in the brain, can quickly and powerfully activate both the inflammatory and immune systems to defend the brain (38). Risk variations of AD that are connected to the microglia of the elderly brain have contributed to an important function for microglia in contemporary AD research, such as TREM2, CD33, INPP5D, HLA-DQA1, and ATXN7L (39). Various studies suggest that inadequate lipid processing and microglial phagocytosis of ab plaques may be at least partially responsible for the disease, even though the exact role of microglia in the process is still unknown (40). In addition, microglia or macrophages can make up to 30–50% of the cells in gliomas (41). Within and surrounding glioma tissue, macrophages and microglial cells proliferate and take on an amoeboid appearance. Microglia can be attracted to glioma cells by the secretion of scatter factor, hepatocyte growth factor and so on (42). It’s possible that glioma survivors’ cells, such as microglia, may have developed a unique phenotype during the illness and therapy that inhibits the progression of AD. Additionally, gliomas are associated with some of the same risk genes for ADs, one example is TREM2 (43). According to GWAS, TREM2 may be essential to the pathophysiology of AD (44). TREM2 may have a variety of roles in microglial processes related to AD brain homeostasis (45). TREM2 can function alone or in conjunction with additional molecules, such as apolipoprotein E (APOE), to affect microglial functions in disorders caused by amyloid and tau, as well as inflammation and metabolism (45–47). Moreover, it was discovered that excessive TREM2 expression in malignant brain tumors is linked to a worse prognosis, while low TREM2 expression is linked to a higher chance of survival (48). It is conceivable that competing genotypes of the same gene are linked to both AD and brain cancer, explaining the seemingly paradoxical results of AD and glioma sharing overexpressed genes but having negatively correlated prevalence. Our research indicates that glioma risk may have a protective effect against AD development and validates its involvement in AD etiology.

While glioma and MS co-occurring is extremely uncommon, reports of such cases have been documented dating back to 1949 (49), which raises the possibility of underlying mechanisms between glioma and MS, perhaps resulting from the environment or heredity. However, whether there exists a causal association and the effect direction remains unknown. Consideration between glioma and MS may be given to the possible involvement of DNA methylation. Glioma growth and progression are significantly influenced by epigenetic changes, which are regarded as a marker (50). DNA methylation, including hypermethylation, hypomethylation across the genome, and hypomethylation specific to certain genes, has been implicated in majority of studies on epigenetic changes in GBM thus far (51). In MS patients, several brain regions have mismethylated genes having a particular profile or low methylation, like the cytosine in the promoter of the myelin enzyme peptidylarginine deiminase-2 in MS-normal-appearing white matter (52). Furthermore, Sahm et al. identified differentially methylated regions (DMRs) involving immune-related genes, such as human leukocyte antigen (HLA) and interleukin regions, by comparative analysis of genome-wide DNA methylation patterns in gliomas occurring in patients with and without MS (53). Additionally, from an immunological point of view, gliomas and MS represent opposing paradigmatic states inside the CNS. Significant immunological abnormalities (54), including as CD4 lymphopenia (55), elevated regulatory T cell percentages in peripheral blood, and changes in cytokine profiles from Th1 to Th2 (56), are present in glioma patients. Whereas overactive immune reactions cause MS. MS is the most prevalent immune-mediated brain illness with radiological features. It is distinguished by axonal damage, severe demyelination, lesion formation in the brain and spinal cord, opening of the blood–brain barrier (BBB), and infiltration of inflammatory immune cells (57). Interestingly, the majority of the recently discovered MS risk genes are immune system-related (58). Meanwhile, germline and somatic immune system changes have been proposed as potential contributors to the pathophysiology of adult glioma in epidemiological investigations (59). Therefore, immune impairment in glioma patients may be a potential mechanism for reducing MS.

Similar to NDs in terms of age range and tissue type, CNS tumors also develop. But there is few epidemiological data about the correlation between NDs and this kind of tumor (60). According to some research, there is a positive correlation between a better prognosis for gliomas and genes linked to both ALS and PD (61). Among these genes, there is the highest association between high Tau/MAPT expression and many markers of longer life in patients with gliomas. Although tau protein has been shown to express in glial cells and gliomas, it controls microtubule dynamics and stability in neurons (62). However, regarding the control of Tau/MAPT transcription in tumors, not much is known (62). In addition, CNS tumors is characterized by a high rate of morbidity and mortality. The majority of these neoplasms develop infrequently, and a number of risk factors, including concurrent illnesses like Parkinson’s disease and exposure to electromagnetic fields or ionizing radiation, have been linked to their formation (63). Most juvenile recessive autosomal cases of PD are caused by Parkin. Parkin and p53’s balance is upset in both PD and brain tumors (64). The significance of the functional interaction between Parkin and p53 is noteworthy, and the pathogenic mutations that disrupt it are probably responsible for the genesis of both PD and gliomas. But it’s still unknown how PD affects the growth of glioma. There was no evidence in our investigation of a causal link between NDs and gliomas. To validate that, more investigation is required.

Current studies showed there is a possible correlation existing between NDs and treatment linked to gliomas. For instance, methylene blue (MB), a medication that has been around for a century, has ability to accept electrons from NADH and transfer them to cytochrome C, offering a different route for electron transfer (65). In glioma treatment, MB reduces glioma proliferation in cells, stops the glioma cell cycle at S-phase, and reverses the Warburg effect by increasing mitochondrial phosphorylation by oxidation (66). A clinical Phase II trial evaluated the effects of MB therapy on cognitive impairment in 332 presumably AD patients were presented (67). Furthermore, several studies have shown that MB protects neurons and astrocytes from a variety of stressors *in vitro* and in rat models of AD and PD (68, 69). Our results suggest that glioma is associated with AD and MS. However, the glioma data used in our MR analysis did not include information on whether patients with glioma received treatment. Further research is necessary to determine how therapies in glioma patients affects NDs.

Our study has several advantages. Firstly, a two-sample bi-directional MR approach was used to draw causal conclusions between glioma and NDs risk while controlling for confounders and reverse causality. Secondly, in comparison to previous research, ours demonstrated robust validity and generalizability since it used data on glioma from the biggest GWAS dataset, comprising 12,488 cases and 18,020 controls, as well as data on NDs from an impartial large-scale GWAS dataset. Thirdly, we incorporated fresh elements. That have not been studied before in previous MR research, such as MS and ALS.

However, it is important to take into account this study’s shortcomings as well. Firstly, due to the lack of data on gender or age stratification, our analysis may be influenced by these factors. Secondly, our study was limited to using European-ancestry whole-genome association data, which may result in limited applicability of our findings to other populations. Nevertheless, our goal is to include all populations in our analysis as much as feasible.

## Conclusion

In summary, our findings showed a genetic correlation between glioma and NDs. Meanwhile, the risk of AD and MS may be lowered by glioma. These results contribute to our understanding of the function of glioma in NDs and will enable the development of therapeutic medications for glioma complications in upcoming clinical trials. In addition, our research sheds further light on the development of NDs and justifies more study to identify the precise pathways underlying their pathophysiology.

## Data availability statement

The datasets presented in this study can be found in online repositories. The names of the repository/repositories and accession number(s) can be found in the article/Supplementary material.

## Author contributions

YL: Conceptualization, Data curation, Formal analysis, Funding acquisition, Investigation, Methodology, Project administration, Resources, Software, Supervision, Validation, Visualization, Writing – original draft, Writing – review & editing. YC: Writing – original draft, Writing – review & editing, Conceptualization, Data curation, Formal analysis, Funding acquisition, Investigation, Methodology, Project administration, Resources, Software, Supervision, Validation, Visualization. MG: Conceptualization, Writing – review & editing. JL: Methodology, Writing – review & editing. YaW: Methodology, Writing – review & editing. YiW: Validation, Writing – review & editing. YG: Investigation, Writing – review & editing. LY: Methodology, Writing – review & editing. JW: Writing – review & editing. NW: Writing – review & editing.
